# Multi‐Omics Insights Into Circulating T‐Cell Metabolic Reprogramming and Treg Dysfunction in Moyamoya Disease

**DOI:** 10.1002/mco2.71088

**Published:** 2026-10-23

**Authors:** Zhiyao Zheng, Qiheng He, Xinyu Ma, Bojian Zhang, Junsheng Li, Chenglong Liu, Siqi Mou, Wei Sun, Xiangjun Shi, Wenjing Wang, Dong Zhang, Peicong Ge, Jizong Zhao

**Affiliations:** ^1^ Department of Neurosurgery Beijing Tiantan Hospital Capital Medical University Beijing China; ^2^ Basic and Translational Medicine Center National Clinical Research Center for Neurological Diseases Beijing Tiantan Hospital Capital Medical University Beijing China; ^3^ Research Unit of Accurate Diagnosis Treatment, and Translational Medicine of Brain Tumors Chinese Academy of Medical Sciences and Peking Union Medical College Beijing China; ^4^ Department of Neurosurgery Peking Union Medical College Hospital Chinese Academy of Medical Sciences and Peking Union Medical College Beijing China; ^5^ Department of Rheumatology and Immunology Beijing Tiantan Hospital Capital Medical University Beijing China; ^6^ Beijing Institute of Hepatology Beijing Youan Hospital Capital Medical University Beijing China; ^7^ Department of Neurosurgery Beijing Hospital National Center of Gerontology Beijing China; ^8^ Institute of Geriatric Medicine Chinese Academy of Medical Sciences Beijing China

**Keywords:** metabolic reprogramming, Moyamoya disease, neuroinflammation, regulatory T‐cell

## Abstract

Moyamoya disease (MMD) is a progressive cerebrovascular disease linked to immune dysregulation, yet T‐cell metabolic reprogramming and regulatory T‐cell (Treg) function remain poorly understood. Here, we integrated multi‐omics approaches, including mass cytometry, single‐cell RNA sequencing, bulk RNA sequencing, metabolomics, and flow cytometry to characterize T‐cell metabolic alterations and Treg dysfunction in MMD, uncovering a key metabolic‐immune axis. Compared with healthy controls, MMD patients showed chronic immune activation and broad suppression of amino acid, glycolytic, and fatty acid metabolic pathways. Although Tregs were aberrantly expanded, they displayed metabolic inflexibility, together with impaired intercellular communication. Single‐cell transcriptomics indicated Treg‐to‐Th17 conversion, while flow cytometry confirmed increased IL‐17A^+^ cells within the CD4^+^FOXP3^+^ compartment. Besides, these dysfunctional Tregs correlated with advanced Suzuki stages, ischemic stroke, and posterior cerebral artery involvement. Bulk transcriptomic and serum metabolomic analyses further confirmed coordinated immune activation and metabolic suppression at the gene, protein, pathway, and metabolite levels. These findings define a metabolic–immune axis in MMD in which metabolically constrained, functionally unstable Tregs may contribute to neuroinflammation and vascular remodeling, suggesting that restoring Treg metabolic fitness could represent a therapeutic strategy.

## Graphical abstract

The comprehensive study design is illustrated. A multi‐omics approach was used to characterize T‐cell metabolic alterations in MMD. By integrating mass cytometry (CyTOF), single‐cell RNA sequencing (scRNA‐seq), bulk RNA‐seq, metabolomics, and flow cytometry, we aimed to elucidate metabolic reprogramming in circulating T cells and Treg dysfunction in MMD patients.

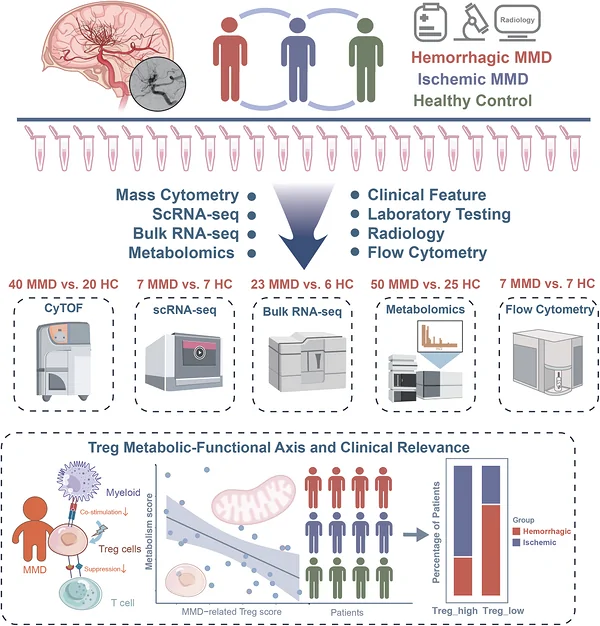

## 1 Introduction

Moyamoya disease (MMD) is a rare, progressive cerebrovascular disorder characterized by stenosis or occlusion of the terminal internal carotid arteries (ICAs) and their major branches, accompanied by the formation of fragile collateral vessels [1, 2]. Without timely intervention, MMD can lead to life‐threatening stroke events and impose a serious economic burden on families and society [3]. Despite advancements in surgical revascularization, including direct bypass and indirect bypass, the long‐term outcomes remain disappointing due to the limited knowledge of the mechanisms driving disease progression and recurrence [4]. In recent years, accumulating evidence has shifted from purely vascular etiologies toward immune‐mediated dysregulation in MMD pathophysiology [5]. Studies have reported infiltration of inflammatory cells, including T lymphocytes, into the walls of abnormal arteries, along with elevated and altered pro‐inflammatory cytokines (e.g., interleukins and C‐X‐C motif chemokines) in peripheral blood and cerebrospinal fluid (CSF) [4, 6]. These findings underscore the potential role of adaptive immunity, particularly T‐cells, in driving vascular inflammation and remodeling in MMD.

T cells rely on dynamic metabolic reprogramming to support activation, proliferation, and function [7]. Upon antigen stimulation, naive T‐cells transition from quiescent, oxidative phosphorylation (OXPHOS)‐dominant metabolism to aerobic glycolysis and glutaminolysis, upregulating enzymes such as lactate dehydrogenase A (LDHA) and glutaminase (GLS) [7]. Differentiation into effector or regulatory subsets further polarizes metabolic pathways: Th17 cells rely on glycolysis and fatty acid synthesis, while regulatory T‐cells (Tregs) prefer OXPHOS and fatty acid oxidation (FAO) to maintain long‐term survival and suppressive function [8]. Disruptions in these metabolic programs can impair T‐cell functionality, contributing to either excessive inflammation or defective regulation, both of which may exacerbate tissue damage in inflammatory diseases. However, the metabolic profiles of T cells in MMD, particularly Tregs, remain largely unknown. In our prior work, we suggested altered Treg distribution in MMD pathogenesis [9]. Yet, it is unclear whether these Treg changes are linked to specific metabolic alterations, or how their metabolism may be reprogrammed in MMD. These gaps limit our understanding of the immune dysfunction driving this disease.

In this study, we utilized a multi‐omics approach to characterize T‐cell metabolic alterations in MMD, providing insights into its potential impact on T‐cell function and immune dysregulation. By integrating mass cytometry (CyTOF), single‐cell RNA sequencing (scRNA‐seq), bulk RNA‐seq, and metabolomics, we aimed to elucidate metabolic reprogramming in circulating T‐cells and Treg dysfunction in MMD patients. Our primary objectives were to characterize metabolic phenotypes of T‐cell subsets in MMD, identify metabolic pathways associated with Treg dysfunction, and correlate these metabolic signatures with clinical phenotypes.

## 2 Results

### 2.1 Aberrant T‐Cell Proportion and Metabolic Feature in PBMCs of MMD Patients

To determine the metabolic phenotype of T‐cell subsets in MMD patients, we performed immune and metabolic profiling in a total of 40 MMD patients and 20 healthy controls (HCs, Table S1). T cells were gated from PBMC (Figure S1), and the subpopulations of CD4 T cell, CD8 T cell, double‐negative T cell (DNT), and double‐positive T cell (DPT) were identified after clustering (Figure 1A,B). Across distinct T‐cell subpopulations, we observed diverse states in terms of identity, memory, differentiation, activation, and effector function (Figure 1C). Compared to HCs, MMD patients exhibited higher levels of CCR7 and CD45RO, but lower CD45RA, indicating a shift from naïve to memory‐dominant phenotype (Figure 1D). This pattern suggests that T cells in MMD patients have undergone antigen‐driven activation and differentiation. In addition to these differentiation markers, HLA‐DR, an MHC class II molecule and activation marker, was significantly upregulated in T cells from MMD patients, further supporting enhanced immune activation (Figure 1D). Conversely, the co‐inhibitory molecules CD152 and CD279, which are critical for maintaining immune tolerance and preventing excessive activation, showed significant downregulation, indicating impaired inhibitory signaling. Meanwhile, the co‐stimulatory molecule CD7, which is essential for T‐cell survival, was also significantly decreased in MMD patients, potentially reflecting impaired co‐stimulatory signaling and effector dysfunction. Collectively, these alterations suggest that T cells in MMD exist in a chronically activated yet dysregulated effector state, which may contribute to immune imbalance and the pathogenesis of the disease.

**FIGURE 1:**
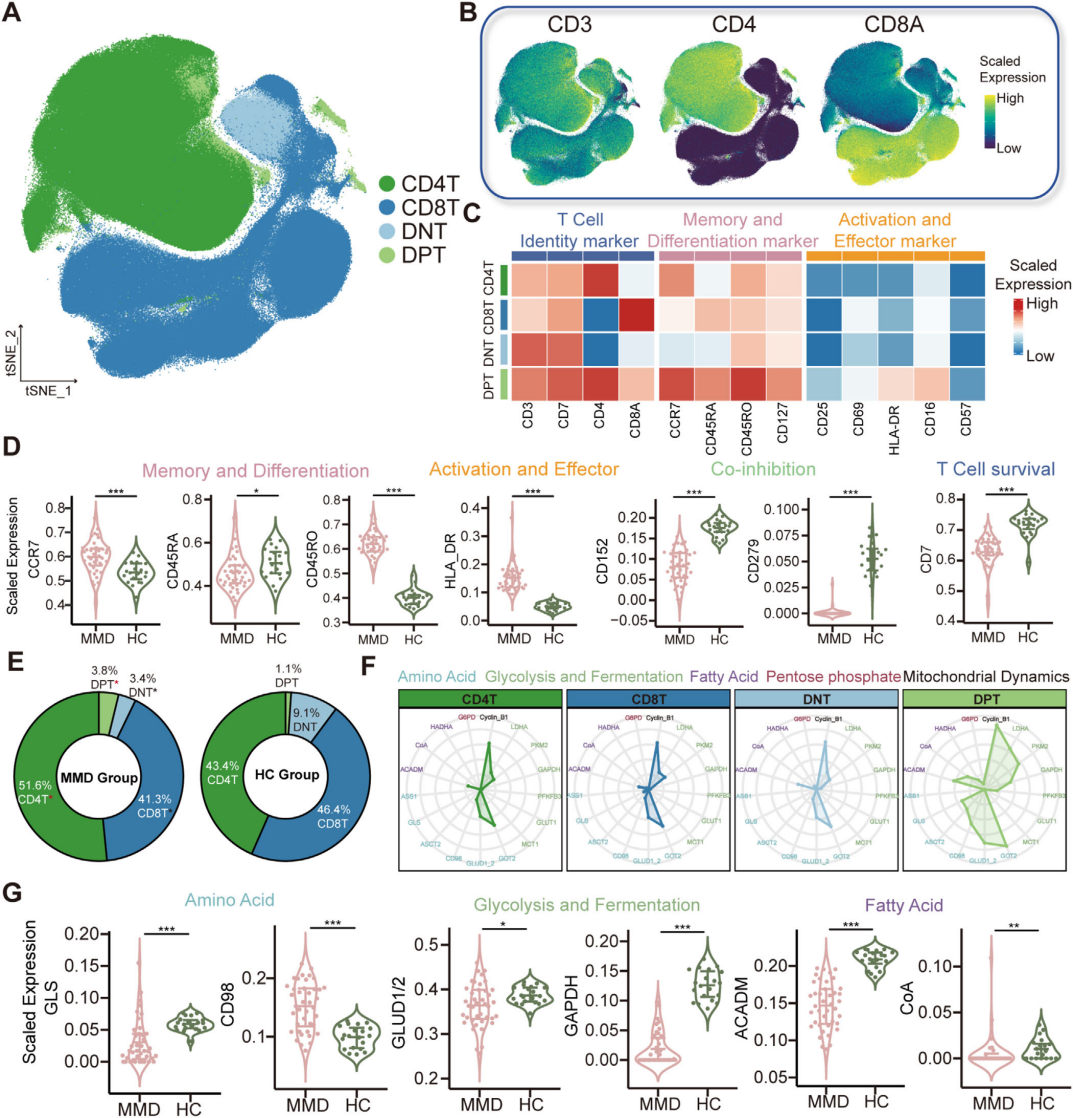
Aberrant T‐cell proportion and metabolic features in PBMCs of MMD patients. (A) t‐distributed stochastic neighbor embedding (t‐SNE) plot, colored by major cell types. (B) t‐SNE plot of typical representative markers in each subpopulation, with expression levels distinguished by color. (C) Heatmap showing the distinct marker expression in each subpopulation. The color represents scaled expression level among different groups. (D) Violin plots showing the differentially expressed immune markers in moyamoya disease (MMD) patients (*N* = 40) compared with healthy controls (HCs, *N* = 20). Mann–Whitney *U* test, *p < 0.05, **p < 0.01, ***p < 0.001. (E) Pie charts showing relative proportions of T subpopulations in MMD patients (*N* = 40) versus HCs (*N* = 20). Asterisk (*) in red: significantly upregulated; asterisk (*) in black: significantly downregulated. Mann–Whitney *U* test, *p < 0.05. (F). Radar chart showing the expression levels of markers in metabolic pathways in different T‐cell subpopulations. (G). Violin plots showing the differentially expressed metabolic markers in MMD patients (*N* = 40) compared with HCs (*N* = 20). Mann–Whitney *U* test, *p <0.05, **p < 0.01, ***p < 0.001. DNT, double‐negative T‐cell; DPT, double‐positive T‐cell; HC, healthy control; MMD, moyamoya disease.

Regarding the abundance of distinct T‐cell subpopulations, we observed a significant increase in CD4 T cells (51.6% vs. 43.4%) and a corresponding decrease in CD8 T cells (41.3% vs. 46.4%) in MMD patients compared with HCs (Figure 1E). To explore potential metabolic links, we compared the metabolic profiles of the four subpopulations, which revealed activity across amino acid, glycolysis, and fatty acid metabolism pathways (Figure 1F). We then analyzed the expression of key metabolism‐related markers in MMD versus HCs (Figure 1G). In terms of amino acid metabolism, GLS expression was significantly downregulated, whereas CD98 was significantly upregulated in MMD patients. Regarding glycolysis, GLUD1/2 and GAPDH were both significantly downregulated. For fatty acid metabolism, ACADM and CoA also showed significant downregulation. These findings suggest that T cells in MMD patients undergo metabolic reprogramming, characterized by dysregulation in amino acid, glycolytic, and fatty acid metabolic pathways.

### 2.2 Immune and Metabolic Profiles of CD4 T‐Cell and CD8 T‐Cell Subsets in MMD

Considering the distinct functions and metabolic demands of T‐cell subsets, we further subdivided CD4 T cells and CD8 T cells based on surface markers. CD4 T cells were classified into six subsets, including naive (Tn), central memory (Tcm), effector memory (Tem), regulatory (Treg), terminal effector memory (Temra), and exhausted (Tex) T cells (Figure 2A,B). In MMD patients, CD45RO expression was significantly increased, whereas activation and effector molecules such as CD25, CD69, HLA‐DR, CD16, and CD57 were significantly downregulated (Figure 2C). This suggests that CD4 T cells in MMD patients exist in a state of imbalanced immune activation, characterized by memory skewing, sustained antigenic stimulation, and impaired effector function. Regarding subset proportions, we found that the proportion of CD4 Tcm was significantly increased, while the proportion of CD4 Tn and CD4 Tex were decreased (Figure 2D,E, Figure S2A). Interestingly, among CD4 T cells, the proportion of Tregs was also significantly upregulated (Figure 2E). However, it remains unclear whether the increased number of Tregs corresponds to normal immunosuppressive function. Considering the overall immune activation imbalance observed in MMD, these expanded Tregs may be functionally impaired, warranting further investigation into their phenotype and metabolism.

**FIGURE 2:**
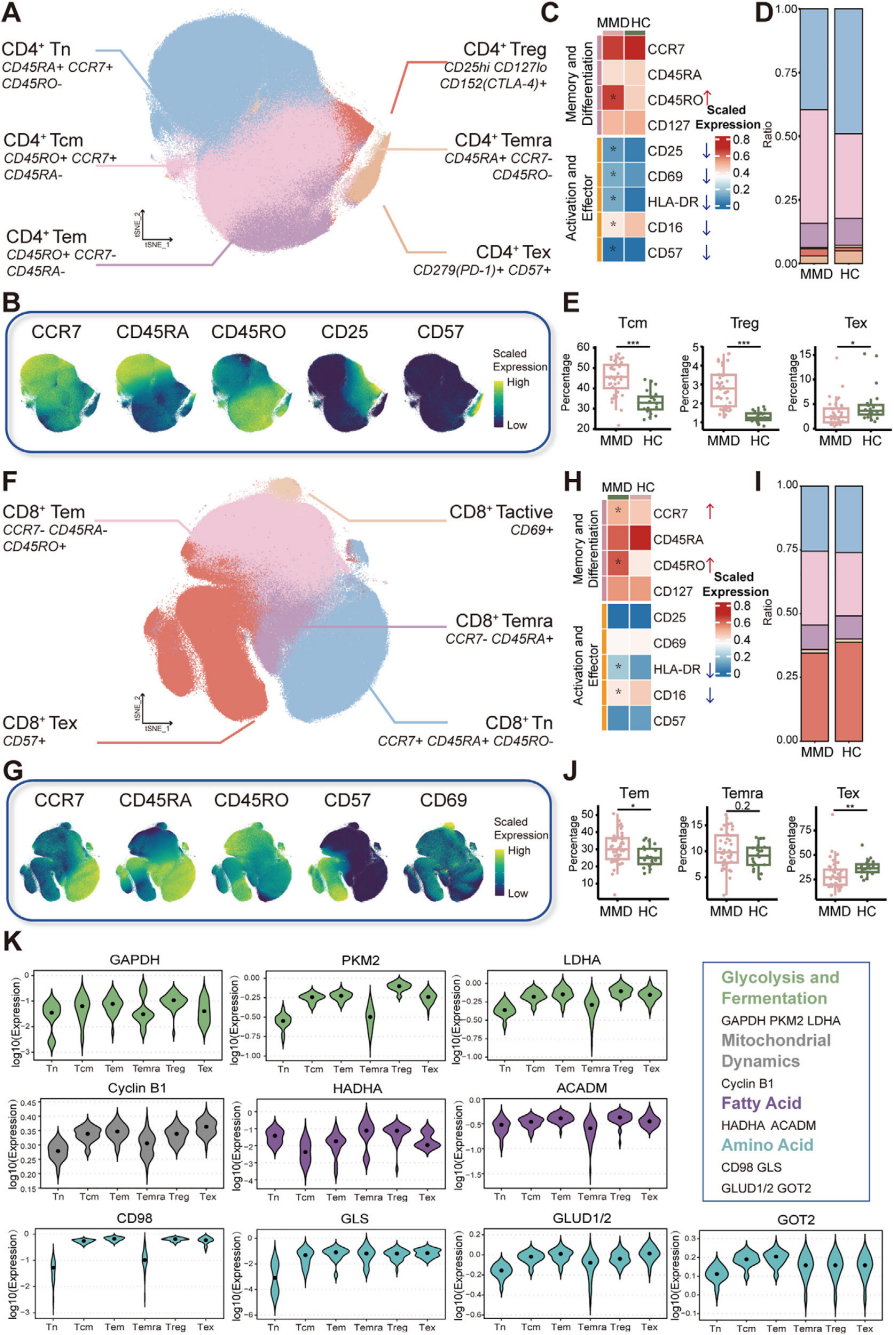
Immune and metabolic profiles of CD4 T‐ and CD8 T‐cell subsets in MMD. (A) t‐distributed stochastic neighbor embedding (t‐SNE) plot of aggregated CD4 T‐cells, colored by major cell types. (B) t‐SNE plot of typical representative markers in each CD4 T‐cells subset, with expression levels distinguished by color. (C) Heatmap showing the differentially expressed immune markers in CD4 T‐cells in moyamoya patients (MMD, *N* = 40) compared to healthy controls (HCs, *N* = 20). The color represents scaled expression level between the two groups. Among the significantly differentially expressed markers, those that are downregulated in MMD compared with HCs are indicated by blue downward arrows, otherwise by red upward arrows. Mann–Whitney *U* test, *p < 0.05. (D) Bar plots showing the CD4 T‐cell subset proportions in MMD patients and HCs. (E) Box plots showing the percentage of CD4 T‐cell subsets in MMD patients (*N* = 40) versus HCs (*N* = 20). Mann–Whitney *U* test, *p <0.05, **p < 0.01, ***p < 0.001. (F) t‐distributed stochastic neighbor embedding (t‐SNE) plot of aggregated CD8 T cells, colored by major cell types. (G) t‐SNE plot of typical representative markers in each CD8 T‐cells subset, with expression levels distinguished by color. (H) Heatmap showing the differentially expressed immune markers in CD8 T cells in MMD patients (*N* = 40) compared to HCs (*N* = 20). The color represents scaled expression level between two groups. Among the significantly differentially expressed markers, those that are downregulated in MMD compared with HCs are indicated by blue downward arrows, otherwise by red upward arrows. Mann–Whitney *U* test, *p < 0.05. (I) Bar plots showing the CD8 T‐cell subsets in MMD patients and HCs. (J) Box plots showing the percentage of CD8 T‐cell subsets in MMD patients (*N* = 40) versus HCs (*N* = 20). Mann–Whitney *U* test, *p < 0.05, **p < 0.01, ***p < 0.001. (K) Violin plots showing the log10‐transformed expression levels of each metabolism marker in different CD4 T‐cell subsets in MMD patients. Tn, naive T cell; Tcm, central memory T cell; Tem, effector memory T cell; Treg, regulatory T cell; Temra, terminal effector memory T cell; Tex, exhausted T cell; Tactive, active T cell.

Besides, we divided CD8 T‐cells into five subsets, including Tn, Tem, Tactive, Temra, and Tex cells (Figure 2F,G). In MMD patients, the expression levels of CCR7 and CD45RO were upregulated in CD8 T cells, while HLA‐DR and CD16 were downregulated (Figure 2H). Subset analysis revealed a significant increase in CD8 Tem and a decrease in CD8 Tex cells (Figure 2I, J, Figure S2B). The results suggest that chronic antigen‐stimulated CD8 T cells in MMD patients undergo a shift toward a memory phenotype.

To investigate the metabolic characteristics of distinct T‐cell subsets in MMD patients, we analyzed the expression levels of key metabolic markers in CD4 T‐cell and CD8 T‐cell subpopulations. Different CD4 T‐cell subsets in MMD patients exhibited distinct metabolic marker expression profiles (Figure 2K). Similarly, CD8 T‐cell subsets also showed subset‐specific metabolic patterns (Figure S3). We further compared the metabolic marker expression between MMD patients and HCs across various CD4 and CD8 T‐cell subsets (Figures S4 and S5). MMD patients displayed significant alterations in metabolic marker expression in both CD4 and CD8 T‐cell subpopulations compared with HCs, further supporting the presence of metabolic reprogramming in T cells from MMD patients.

### 2.3 Treg Cell Proportion and Metabolic Changes Are Closely Related to Clinical Manifestations

Previous studies have suggested that Tregs may increase in MMD but exhibit reduced stability and function [9]. Consistent with this, we demonstrated a significant increase in Treg proportions in MMD patients, alongside heightened sensitivity to metabolic changes. We investigated Treg‐specific metabolic alterations (Figure 3A) and their potential link to dysfunction and clinical features. Tregs from MMD patients showed increased expression of memory and differentiation markers like CCR7, CD45RA, and CD45RO (Figure 3B). Regarding activation and effector markers, we observed a significant upregulation of CD25, CD69 and CD57, alongside a marked downregulation of HLA‐DR and CD16. This discordant expression pattern suggests that Tregs in MMD patients exhibit functional dysregulation, which may compromise their role in maintaining immune tolerance and contributing to the immune imbalance characteristic of MMD. Metabolically, Tregs displayed widespread deficiencies: glycolysis and fermentation markers (GAPDH, LDHA, and PKM2), fatty acid metabolism indicators (ACADM, CoA, and HADHA), and amino acid metabolism (ASCT2, GLS, GLUD1/2, and GOT2) were all significantly downregulated (Figure 3C). CD98 and mitochondrial cyclin B1 showed no change. These findings suggest that despite increased numbers, Tregs in MMD exhibit metabolic insufficiency across multiple pathways, potentially undermining their immunosuppressive function and contributing to immune dysregulation in the disease.

**FIGURE 3:**
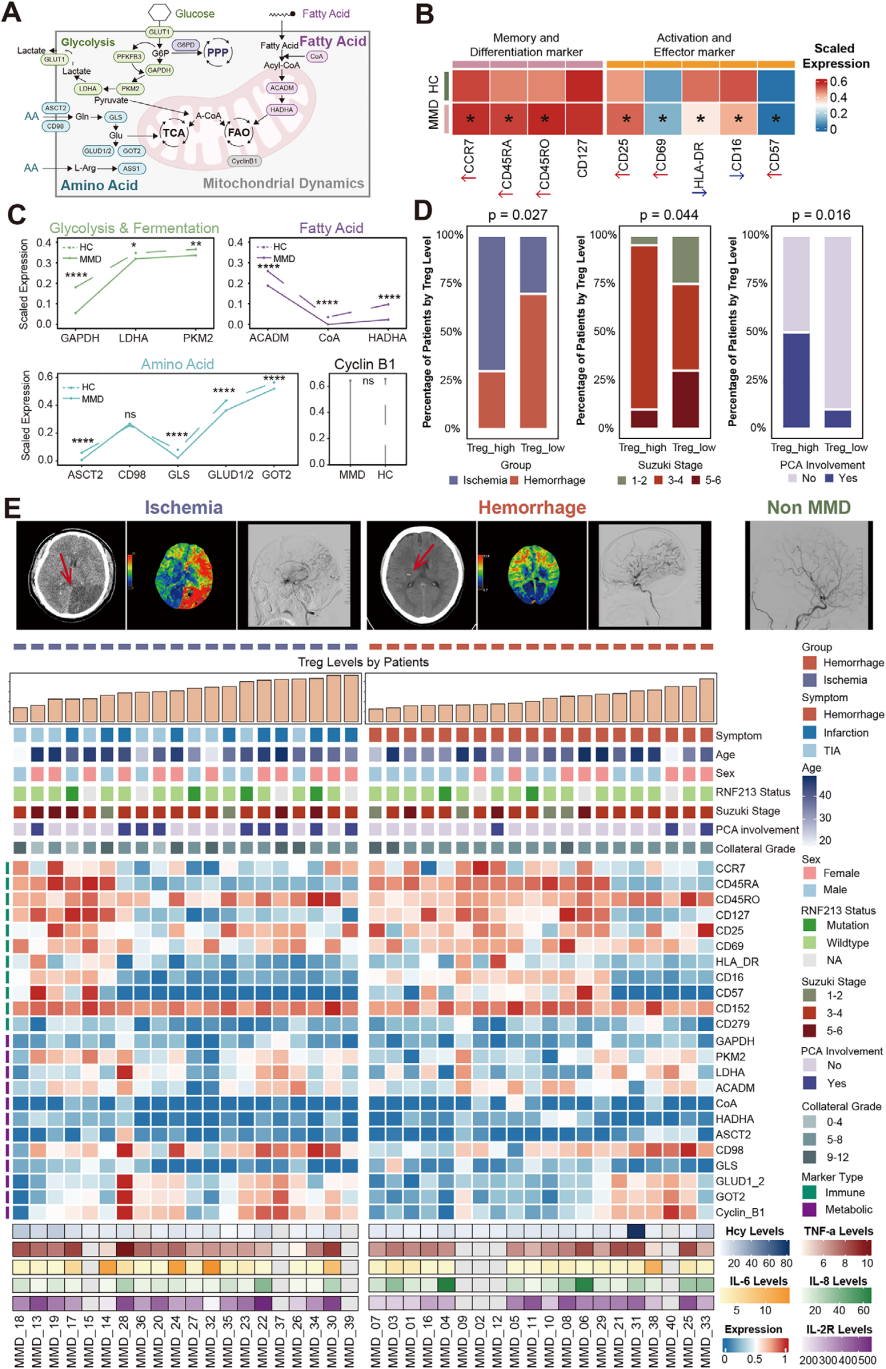
Treg cell proportion and metabolic changes are closely related to clinical manifestations. (A) Conceptual overview of the markers directly measured by mass cytometry. (B) Heatmap showing the differentially expressed immune markers in Tregs in moyamoya (MMD) patients (*N* = 40) compared with healthy controls (HCs, *N* = 20). The color represents scaled expression level between two groups. Among the significantly differentially expressed markers, those that are downregulated in MMD compared with HCs are indicated by blue rightward arrows, otherwise by red leftward arrows. Mann–Whitney *U* test, *p < 0.05. (C) Line graph showing the metabolic markers in Tregs in MMD and HCs. *p < 0.05, **p < 0.01, ***p < 0.001,****p < 0.0001. (D) Bar plots showing the proportion of MMD patients (*N* = 40) in stroke phenotype (left panel), Suzuki stage (middle panel), and PCA involvement (right panel) according to the level of Treg numbers. (E) The upper panel shows clinical images (CT scan, Time to Peak image, and lateral cerebral angiography) according to ischemic type, hemorrhagic type, and non‐MMD patients. The following panel shows the heatmap of patients’ clinical information, immune markers, metabolic markers, and the expression of inflammatory factors. PCA, posterior cerebral artery; TIA, transient ischemic attack.

MMD patients may present with ischemic stroke or hemorrhagic stroke, manifesting as transient ischemic attack (TIA), infarction, or hemorrhage [10, 11]. To assess clinical correlations with Treg levels, patients were stratified into Treg‐high and Treg‐low groups. Notably, the Treg‐high group had a significantly higher incidence of ischemic stroke (Figure 3D). Subsequently, univariate and multivariate logistic regression analyses were performed to identify risk factors for ischemic MMD (Table S5). The results showed that high Treg levels remained significantly associated with an increased risk of ischemic stroke, indicating that elevated Treg level is an independent risk factor for ischemic MMD. Besides, the Suzuki staging system is the most widely used clinical staging for MMD patients, which is mainly used to evaluate the progression of vascular lesions, guide treatment decisions, and predict postoperative patient outcomes [12, 13, 14]. We found that the distribution of Suzuki stages differed significantly between Treg‐high and Treg‐low groups in MMD patients, suggesting that elevated Treg levels may be associated with a poorer prognosis (Figure 3D). Furthermore, it is reported that posterior cerebral artery (PCA) involvement may be associated with higher rates of perioperative complications and worsened functional outcomes [14, 15, 16]. We consistently found that nearly 50% of patients in the Treg‐high group had PCA involvement, which was significantly higher than that in the Treg‐low group (Figure 3D). To further explore immune‐metabolic links with clinical subtypes, we compared hemorrhagic and ischemic groups by Treg level (Figure 3E). We found that among the immune markers, activation and effector markers such as CD25, CD69, and CD57 tended to decrease with higher Treg proportions. Besides, when comparing stroke subtypes, these markers were significantly more elevated in hemorrhagic stroke patients than in those with ischemic stroke, suggesting subtype‐specific differences in Treg activation status.

### 2.4 ScRNA‐seq Reveals Enhanced Inflammation in MMD and Functionally Reprogrammed Tregs

To validate CyTOF findings and further explore Treg alterations in MMD, we analyzed scRNA‐seq data from PBMCs of MMD patients and HCs (Table S2). We first manually annotated the major cell types, including myeloid cells, neutrophils, mast cells, plasmacytoid dendritic cells (pDC), T and NK cells, B cells, and platelets (Figure S6A–E). To further characterize T‐cell heterogeneity, we performed dimensionality reduction and clustering. CD4 T cells and CD8 T cells were divided into five subsets, respectively, alongside two NK subsets and one cluster of proliferating T cells (Figure 4A, Figure S6F,G). In CD4 T cells, Tregs specifically expressed CTLA4, FOXP3, IL2RA, and RTKN2 (Figure 4B). Despite high expression of co‐stimulatory markers (CD27, ENTPD1, ICOS, TNFRSF9), Tregs also upregulated exhaustion markers (PDCD1, TOX, TIGIT, HAVCR2), suggesting a functionally reprogrammed and potentially impaired state (Figure 4C). Pseudotime analysis showed that Tregs positioned earlier along the developmental trajectory, while Th17 cells appeared at later stages, indicating possible pro‐inflammatory reprogramming (Figure 4D). Further, transcription factor analysis of Tregs from MMD patients identified ETV7, FOXP3, IKZF2, HOXB7, and RFX5 as key regulators (Figure 4E).

**FIGURE 4:**
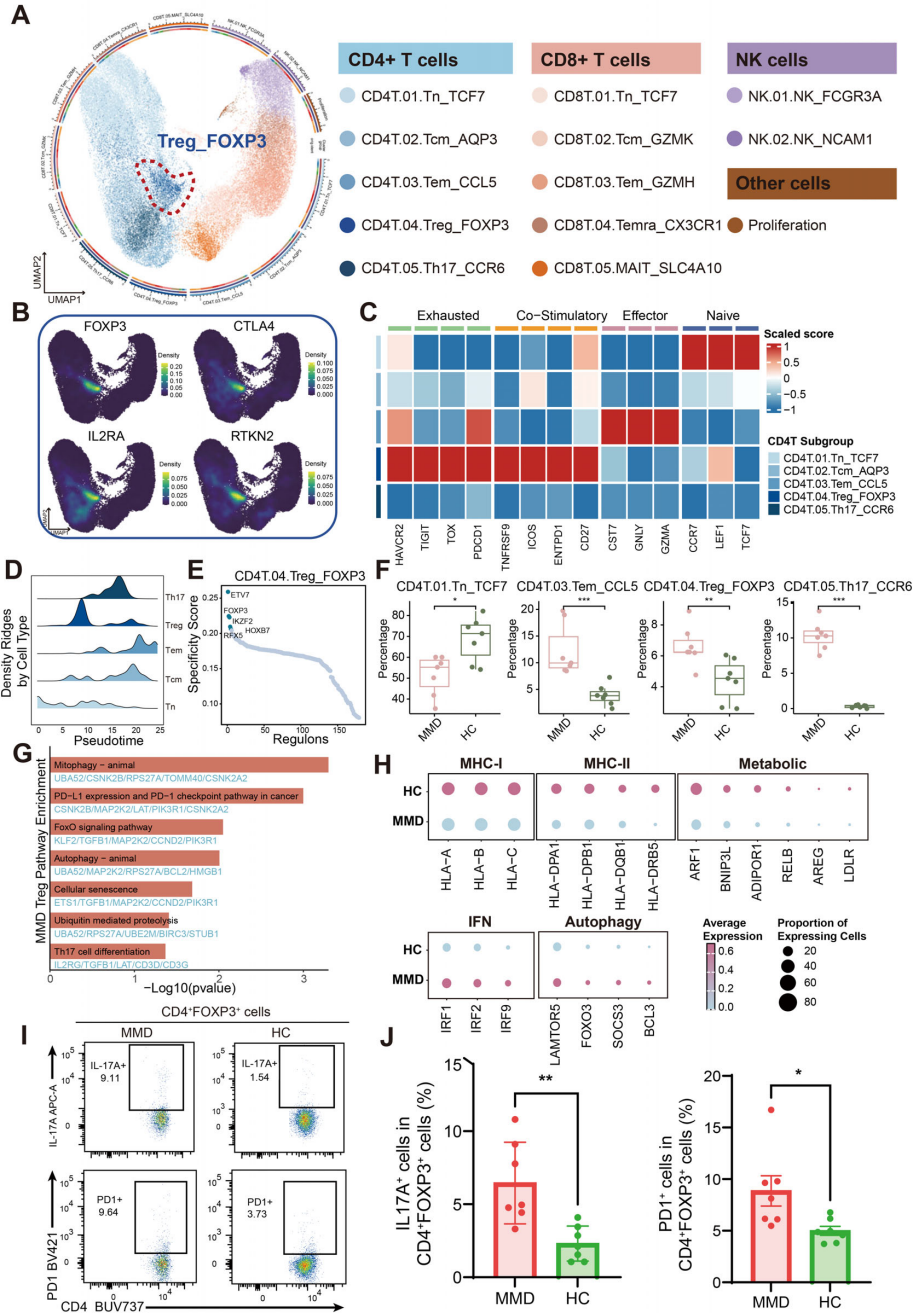
scRNA‐seq reveals enhanced inflammation in MMD. (A) Left panel: Uniform Manifold Approximation and Projection (UMAP) plot, color‐coded by major cell types. The red dotted circle outlines the FOXP3 Treg subset. Right panel: Subsets of CD4 T, CD8 T, NK, and other cells. (B) UMAP plot showing the typical representative genes in each subset, with expression levels distinguished by color. (C) Heatmap showing the differentially markers in each CD4 T‐cell subset. (D) Peak plot showing the pseudotime series of CD4 T‐cell subsets. (E) Dot plot showing the regulon activities in Tregs. (F) Box plots showing the percentage of CD4 T‐cell subsets in MMD patients (*N* = 7) versus HCs (*N* = 7). Mann–Whitney *U* test, *p < 0.05, **p < 0.01, ***p < 0.001. (G) Bar graph showing the KEGG pathways that are significantly enriched in Tregs from MMD patients. (H) Dot plots showing the typical inflammatory and metabolic genes that are differentially expressed in MMD patients compared with HCs. (I) Representative flow cytometry plots showing the expression of IL‐17A^+^ and PD‐1^+^ in CD4^+^FOXP3^+^ Treg cells. (J) Quantification of the proportions of IL‐17A^+^ and PD‐1^+^ in CD4^+^FOXP3^+^ Treg cells in MMD patients (*n* = 7) and healthy controls (*n* = 7). Data are presented as mean ± SEM. Unpaired two‐tailed Student's *t*‐test. *p < 0.05, **p < 0.01, ***p < 0.001. IL‐17A, interleukin‐17; PD‐1, programmed death‐1.

We next compared the composition of CD4 T‐cell subsets between MMD patients and HCs. MMD patients showed a significant decrease in CD4 Tn cells and an increase in CD4 Tem, Th17, and Tregs (Figure 4F). To explore the role of Tregs in MMD, we specifically analyzed their upregulated genes and KEGG enrichment revealed activation of the Th17 cell differentiation pathway, corroborating the pseudotime analysis that suggested a temporal relationship with Tregs preceding Th17 cells (Figure 4G). Additionally, MHC class I and II molecule expression was reduced in MMD Tregs, suggesting impaired antigen responsiveness. Meanwhile, the expression of interferon regulatory factors (IRFs) and autophagy‐related molecules was enhanced, indicating the ability of Tregs to transfer to an unstable, dysfunctional phenotype. In addition, key metabolism‐associated genes, including ARF1, BNIP3L, ADIPOR1, RELB, AREG, and LDLR, were all significantly decreased, indicating that the overall metabolic level of Tregs in MMD patients was decreased (Figure 4H). To further validate the status of Tregs in MMD, we isolated Tregs from PBMCs and performed flow cytometry analysis. The results showed that the proportions of IL‐17A^+^ cells and PD‐1^+^ cells within the CD4^+^ FOXP3^+^ population were increased in MMD patients compared with healthy controls (Figure 4I,J). The above results show that although the number of Tregs in MMD increases, their conversion from a stable to an unstable phenotype weakens their immunosuppressive function, thereby enhancing the inflammatory response in MMD patients.

### 2.5 Disrupted Immune Cell Crosstalk and Regulatory Signaling in MMD Tregs

Previous studies have suggested that the interaction between myeloid cells and Tregs plays an important role in maintaining the immunosuppressive function of Tregs [17, 18]. To explore this relationship in MMD, we examined the correlation between different cell types. Interestingly, we found that myeloid cells and Tregs were negatively correlated in MMD patients, while a positive correlation was identified in HCs (Figure 5A). Consistent with this finding, Cellchat analysis revealed markedly reduced interaction between myeloid cells and Tregs in MMD (Figure 5B, Figure S7). Similarly, communication between Tregs and other T‐cell subsets was also markedly diminished (Figure 5B, Figure S7). These findings suggest that in MMD, Tregs exhibit impaired crosstalk with both antigen‐presenting and effector cells, leading to reduced immunosuppressive capacity and heightened immune dysregulation.

**FIGURE 5:**
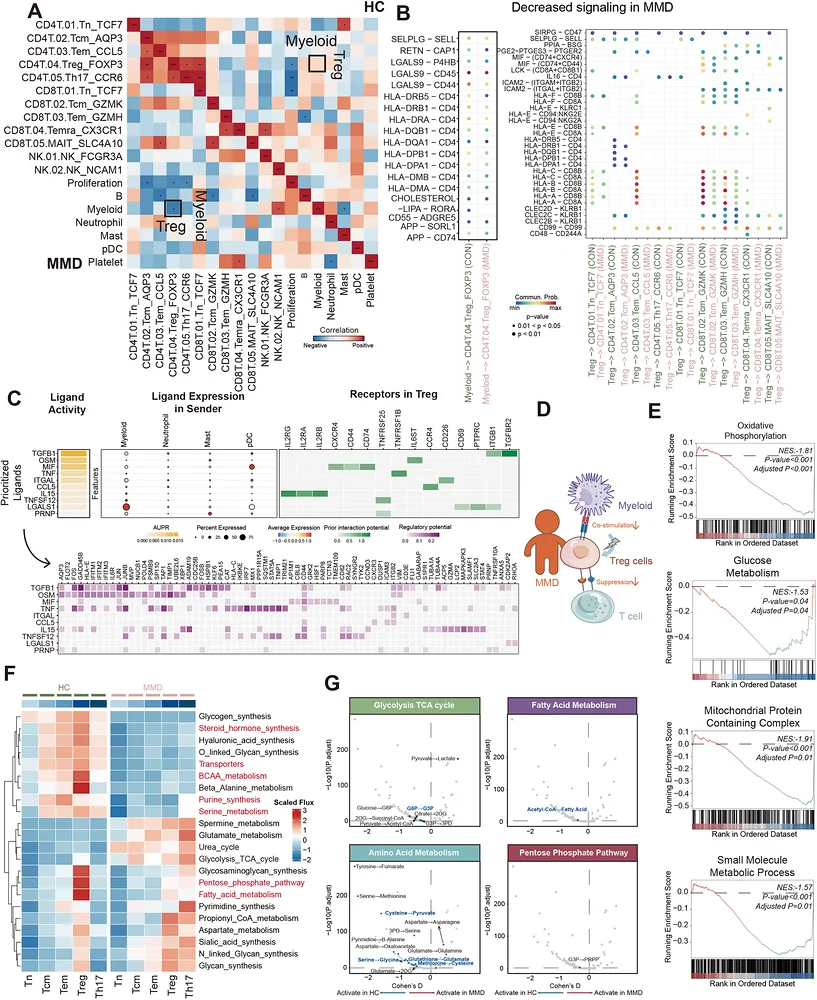
scRNA‐seq reveals attenuated metabolism in functional reprogrammed Tregs in MMD. (A) Heatmap showing the correlation between immune cells in MMD (left, *N* = 7) and HCs (right, *N* = 7). The black box circles the correlation between Tregs and myeloid cells in MMD (left) and HCs (right). Spearman correlation analysis, *p <0.05, **p < 0.01, ***p < 0.001. (B) Dot plots showing the communication probability of ligand‐receptor pairs by CellChat for cell communication from immune cells to Tregs (lower‐left triangle) and from Tregs to immune cells (upper‐right triangle) in MMD and HCs. (C) Ligand activity and intracellular signaling inference in Tregs of MMD patients by NicheNet. Upper left panel: Heatmap showing the ligand activity in sender cells. Upper middle panel: Dot plot showing the ligand expression in different sender cells. Upper right panel: Heatmap showing the receptors in Tregs. Lower panel: Heatmap showing the predicted target genes in Tregs. (D) The schematic diagram showing the weakened ability of Tregs to accept co‐stimulation and exert inhibitory function in MMD patients. (E). The gene set enrichment analysis (GSEA) of metabolic function gene sets in MMD patients compared with HCs. NES, normalized enrichment score. *p* value is adjusted by Bonferroni correction, adjusted *p* < 0.05, significantly different. (F). Heatmap showing the differential metabolic pathway across CD4 T‐cell subsets. (G) Volcano plot showing the metabolic flux that are significantly differentially expressed in each metabolic pathway in MMD patients compared to HCs. Wilcoxon rank‐sum test used to compare flux scores, and calculated Cohen's *d* to estimate the effect size of differences between groups. *p* value is adjusted by Benjamini–Hochberg method, adjusted *p* < 0.05, significantly different.

To explore the molecular mechanisms underlying these alterations, we examined ligand‐receptor interactions and downstream regulatory pathways in Tregs of MMD patients (Figure 5C). Compared with HCs, MMD Tregs exhibited enhanced activity of multiple ligands, including TGFB1, OSM, MIF, TNF, ITGAL, CCL5, IL15, TNFSF12, LGALS1, and PRNP. Specifically, TGFB1 interacts with ITGB1 and TGFBR2 on Tregs. TNF, ITGAL, IL15, TNFSF12, and LGALS1 secreted by myeloid cells may act on TNFRSF1B, CD226, IL2 receptor family, TNFRSF25, and PTPRC on Tregs, respectively. MIF secreted by pDCs may act on CXCR4, CD44, and CD74 of Tregs. According to the predicted target genes, interferon‐related genes such as IFITM1, IFITM2, and IFITM3, and cytoskeleton and extracellular matrix genes such as ITGB2 and VIM in Tregs are regulated by the above ligand receptors. These interactions suggest how impaired immune communication in MMD affects Treg signaling and function (Figure 5D).

### 2.6 Attenuated Metabolism in Functional Reprogrammed Tregs

Given the observed immune and metabolic dysregulation in Tregs, we further analyzed the abnormal metabolic pathways. For Tregs from MMD patients, OXPHOS, glucose metabolism, mitochondrial protein complexes, and small‐molecule metabolic processes were significantly reduced (Figure 5E). Single‐cell flux estimation analysis (scFEA) revealed that Tregs exhibited the highest metabolic heterogeneity among CD4 T‐cell subsets (Figure 5F). Key metabolic processes, including glycogen synthesis, steroid hormone synthesis, transporters, BCAA metabolism, β‐alanine metabolism, purine synthesis, serine metabolism, pentose phosphate pathway, and fatty acid metabolism, were significantly decreased in MMD patients. Notably, metabolic pathways including “G6P→G3P,” “Glucose→G6P,” “Citrate→2OG,” and “Pyruvate→Acetyl‐CoA” in the glycosis TCA cycle, “Acetyl→Fatty Acid” in the fatty acid metabolism, “Tyrosine→Fumarate,” “Serine→Pyruvate,” “Serine→Glycine,” “Glutathione→Glutamate,” and “Methionine→Cysteine” in amino acid metabolism, and “G3P→PRPP” in the pentose phosphate pathway were significantly decreased in MMD (Figure 5G). The results showed that the metabolic level of functionally reprogrammed Tregs in MMD patients was abnormal and characterized by attenuated metabolism.

### 2.7 Reduced Metabolism and Metabolites in MMD Patients From Transcriptome and Untargeted Metabolomics

To further elucidate the immune abnormalities in MMD, we performed bulk RNA‐seq on PBMCs obtained from 23 MMD patients and six healthy controls (Table S3). Through differential gene enrichment analysis, we observed significant enrichment of multiple immune‐related pathways in MMD patients, including the inflammatory response, positive regulation of T‐cell‐mediated cytotoxicity, antigen processing and presentation of exogenous antigen, positive regulation of activated T‐cell proliferation, and interleukin 8 production, confirming the abnormal activation of immune responses in MMD patients (Figure 6A). Of note, in an independent external bulk dataset, we examined the expression of the MMD‐related Treg score in the superficial temporal artery (STA) of MMD patients versus controls, and found that the score was significantly elevated in MMD patients (Figure 6B). This suggests that this abnormal Treg response is not limited to the peripheral immune system but may further infiltrate the diseased vascular wall, thereby contributing to vascular pathology and promoting the progression of MMD.

**FIGURE 6:**
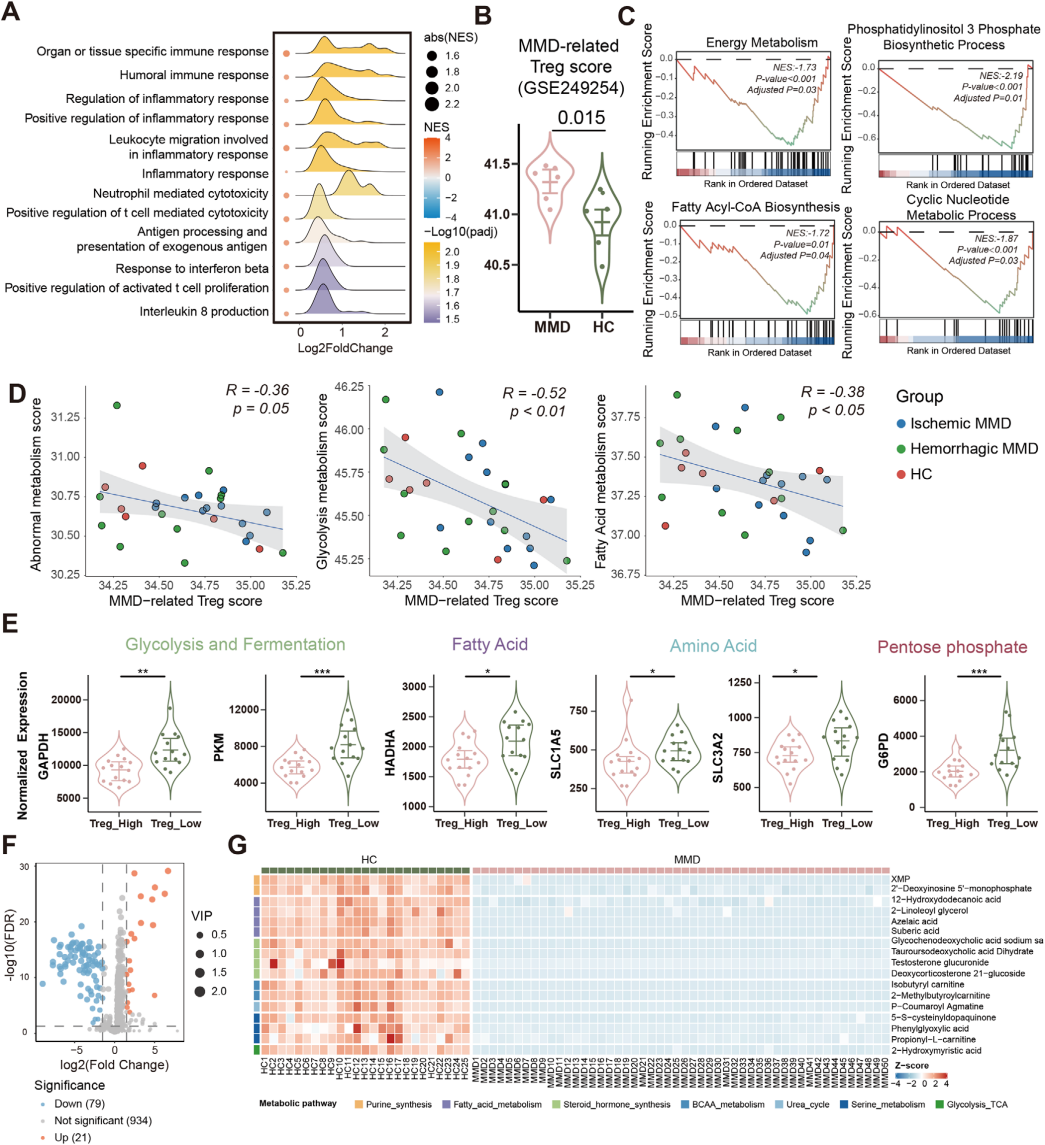
Reduced metabolism and metabolites in MMD patients. (A) Peak plot showing the gene set enrichment analysis (GSEA) of gene sets in moyamoya (MMD) patients (*N* = 23) compared to healthy controls (HCs, *N* = 6). X‐axis showing the Log2FC trend of the gene set, and the bubble color indicates the normalized enrichment score (NES). *p* value is adjusted by Bonferroni correction, adjusted *p* < 0.05, significantly different. (B) Violin plots showing MMD‐related Treg scores in MMD patients and controls in bulk RNA‐seq (GSE249254). (C) Graph showing the comparative enrichment analysis of metabolic function gene sets in MMD patients (*N* = 23) compared with HCs (*N* = 6) from bulk RNA‐seq. NES, normalized enrichment score. *p* value is adjusted by Bonferroni correction, adjusted *p* < 0.05, significantly different. (D) The dot plots showing the Pearson correlation analysis between MMD‐related Treg scores and metabolic scores in MMD patients (*N* = 23) and HCs (*N* = 6). *p* < 0.05, significantly different. (E) Violin plots showing the expression of metabolism‐related markers in the Treg‐high and Treg‐low groups. Mann–Whitney *U* test, *p < 0.05, **p < 0.01, ***p < 0.001. (F) Volcano plot showing significantly differentially expressed metabolites in MMD patients (*N* = 50) and HCs (*N* = 25). Blue, down‐regulated metabolites; red, up‐regulated metabolites. VIP, variable importance in projection. Student's *t*‐test, metabolites were considered significantly different if they met the following criteria: VIP > 1, FDR < 0.05, and |log_2_fold change| > 1.5. (G) Heatmap showing the expression of key metabolites in MMD patients (*N* = 50) and HCs (*N* = 25). FDR, false discovery rate; VIP, variable importance in projection.

Given these immune alterations, we next sought to further validate the metabolic disturbances in MMD. To this end, we performed GSEA analysis on our bulk RNA‐seq data using metabolic gene sets. Interestingly, we noted that pathways related to energy metabolism, fatty acid CoA biosynthesis, cyclic nucleotide metabolism, and phosphatidylinositol 3 phosphate biosynthetic processes were significantly decreased in MMD patients (Figure 6C). We then correlated the degree of these metabolic reductions with the MMD‐related Treg score and found that glycolysis metabolism and fatty acid metabolism showed a significantly negative correlation with the increase of MMD‐related Treg score (Figure 6D). Furthermore, we verified the differential protein changes observed in the CyTOF data. Specifically, we found the glycolytic metabolism genes GAPDH and PKM, the fatty acid metabolism gene HADHA, the amino acid metabolism genes SLC1A5 (encoding ASCT2), SLC3A2 (encoding CD98), and the pentose phosphate metabolism gene G6PD were significantly decreased in the high Treg group (Figure 6E). The results suggest that these metabolic genes may serve as biomarkers for predict Treg abundance in MMD patients.

At the metabolomics level, we found that there were 100 differentially expressed metabolites in MMD patients, of which 79 metabolites were downregulated and only 21 metabolites were upregulated, indicating a notable metabolic downregulation (Figure 6F). Interestingly, the downregulated metabolites showed a strong agreement with the predicted metabolic reductions in MMD from the scRNA‐seq‐based scFEA metabolic flux analysis. Among the significantly downregulated metabolites, 17 metabolites were related to scFEA pathways such as purine synthesis, fatty acid metabolism, steroid hormone synthesis, BCAA metabolism, urea cycle, serine metabolism, and glycolysis TCA (Figure 6G). These results indicate that immune and metabolic genes, functional proteins, metabolic pathways, and metabolites are significantly abnormal in Tregs of MMD patients, manifested as abnormal immune activation and decreased metabolic levels.

## 3 Discussion

In this study, we employed a multi‐omics approach, combining CyTOF, scRNA‐seq, bulk RNA‐seq, and untargeted metabolomics, to systematically characterize the peripheral T‐cell immune and metabolic landscape in MMD patients. We identified heightened immune activation across T‐cell subsets, with expanded yet functionally impaired Tregs. Our study reveals a previously unrecognized metabolic‐immune axis in MMD, characterized by downregulated amino acid, glycolytic, and fatty acid pathways in Treg cells. This represents the first comprehensive characterization of Treg metabolic reprogramming in MMD patients. These metabolically dysregulated Tregs exhibit reduced suppressive capacity and functional instability, which may contribute to pathogenic immune activation. Importantly, we found a strong correlation between the abundance of unstable Tregs and clinical disease severity, supported consistently across transcriptomic, proteomic, and metabolic levels. These multi‐dimensional associations advance our understanding of how immune metabolism contributes to MMD pathophysiology and highlight their important roles in vascular inflammation and their potential as promising therapeutic candidates.

CyTOF analysis revealed a paradoxical state of immune activation. Although Tregs are increased in number, they are functionally unstable and fail to maintain normal immunosuppressive activity. The phenomenon, previously described as the Treg paradox by Schnell et al., may reflect the poor adaptation of Tregs to ongoing damage and chronic inflammation [19]. Notably, this inadequate suppressor state is not unique to MMD, as similar Treg dysfunction has been observed in other chronic inflammatory conditions such as rheumatoid arthritis and ankylosing spondylitis. Consistently, our scRNA‐seq and bulk RNA‐seq data corroborate that T‐cells in MMD patients prioritize survival over effector function, potentially due to chronic inflammation exposure and microenvironmental constraints.

Mechanistically, chronic hypoperfusion and recurrent ischemic or hemorrhagic insults create a hypoxic, inflamed vascular microenvironment that imposes stress on immune cells, leading to a compensatory expansion of Tregs. Additionally, abnormal hemodynamic forces and ongoing vascular injury may promote the release of damage‐associated molecular patterns (DAMPs) and pro‐inflammatory cytokines, further destabilizing Tregs and impairing their suppressive capacity. Tregs exhibit significant phenotypic plasticity, allowing them to adapt to various environmental conditions [20]. However, under certain circumstances, they may lose suppressive capacity and adopt pathogenic features. Our scRNA‐seq data revealed abnormal transcription patterns in Tregs of MMD patients, marked by a shift toward pro‐inflammatory transcriptional reprogramming. This was manifested by the upregulation of interferon‐related and autophagic genes, which have been previously linked to unstable Treg phenotypes [21, 22]. Additionally, enrichment analysis showed activation of the FOXO pathway in MMD Tregs, which partially accounts for reduced IFN‐γ secretion and impaired suppressive function, suggesting a potential shift toward a Th1‐like Treg phenotype [23]. Moreover, the Treg‐Th17 balance is critical in various immune diseases [24, 25], and our pseudotime trajectories revealed a propensity for Treg‐Th17 conversion in MMD. Additionally, disrupted ligand‐receptor interactions impaired Treg communication with other cells, disrupting immunoregulatory networks. Collectively, these findings indicate that Tregs in MMD patients undergo profound phenotypic and functional alterations, characterized by transcriptional instability, lineage shifting, and impaired intercellular communication, which may collectively contribute to the breakdown of immune homeostasis in this disease.

Beyond peripheral immune dysregulation, we found that the MMD‐related Treg score was also elevated in the vascular tissues of MMD patients, suggesting that these cells may aggregate within diseased vessels and further contribute to tissue injury. Indeed, dysfunctional Tregs have been shown to affect vascular function by modulating the structural and metabolic properties of endothelial cells, which in turn affects vascular integrity and the recruitment or migration of other immune cells [26]. Specifically, Tregs can regulate endothelial cell adhesion molecules, intercellular junctions, and chemokines, thereby influencing the trans‐endothelial migration of immune cells [27]. These processes may contribute to the pathogenesis and progression of MMD. Clinically, the increased frequency of Tregs in MMD patients correlates with more advanced Suzuki stages and an elevated incidence of ischemic strokes, suggesting that these dysfunctional Tregs may contribute to disease progression, confirming our hypothesis [28].

T cells will undergo metabolic reprogramming in response to the microenvironment and their own demands, mediating immune responses through multiple pathways [29]. Previous studies demonstrated that during chronic infection or tumor progression, T cells exhibit global metabolic repression despite activation markers, reflecting a state of “metabolic exhaustion” rather than activation [30]. Given the distinct metabolic requirements of T‐cell subsets, we further examined metabolic differences among them and found that Tregs were particularly sensitive to metabolic disruption. Tregs in MMD exhibited significantly impaired glycolysis, fatty acid oxidation, and amino acid metabolism. Glycolysis restriction enhances Treg proliferation but impairs migratory capacity [31, 32], while fatty acid metabolism is critical for Treg suppressive function [33, 34]. Additionally, downregulation of amino acid metabolism has also been reported to lead to a reduction in Treg suppressive function [35]. Notably, despite elevated Treg cell counts in MMD patients, key metabolic pathways such as glycolysis, amino acid, and fatty acid were attenuated, indicating a decoupling between immune and metabolic support. This metabolic‐phenotypic mismatch points toward a metabolically exhausted or functionally restrained T‐cell state in MMD. This was further supported by untargeted metabolomics, which revealed decreased levels of metabolites involved in fatty acid and amino acid metabolism, reduced purine synthesis intermediates, and diminished antioxidants, mirroring the pathway suppression observed in scRNA‐seq.

Our work suggests that Treg dysfunction is a tractable therapeutic target. Preclinical studies support this concept, showing that metformin stabilizes Tregs and reduces atherosclerosis in mice [36]. Interestingly, our previous research has shown that statins may be beneficial for MMD patients [37]. Statins have also been reported to regulate Treg function [38, 39], but the results have not yet been verified in MMD patients. Early intervention may delay vascular remodeling and improve postoperative outcomes, particularly in patients who have already been in the high Suzuki stage.

Our study has several limitations. The cross‐sectional design precludes causal inference. Longitudinal cohorts are needed to assess the dynamic changes in immune and metabolic profiles during disease progression. Second, our conclusions regarding Treg functional impairment and metabolic downregulation in MMD are primarily based on transcriptomic and metabolomic signatures. Although limited in vitro experiments have been performed to validate Treg dysfunction in MMD, the relationship between Treg dysfunction and metabolic alterations requires further confirmation through additional in vivo and in vitro studies. Third, our analysis was performed primarily on peripheral blood, meaning that the relationship between peripheral immune findings and intracranial vascular pathology is inferred rather than directly demonstrated. Future studies incorporating intracranial tissue samples, along with immunofluorescence, spatial transcriptomics, and metabolomics, would help refine our understanding of tissue‐specific metabolic niches and Treg behavior at the diseased vascular wall. Fourth, the scRNA‐seq cohort sample size remains limited for rare cell state analysis. Future studies with larger scRNA‐seq cohorts are needed, and we are actively expanding our single‐cell cohort in ongoing research.

## 4 Conclusion

Our work uncovers a metabolic‐immune axis in MMD centered on Treg cell metabolic reprogramming and conversion into an unstable state. By integrating multi‐omics, including CyTOF, scRNA‐seq, bulk RNA‐seq, and untargeted metabolomic data, we identify an association between metabolic pathway alterations and Treg dysfunction in neurovascular inflammation. These findings may advance our understanding of MMD pathobiology and suggest that Treg‐targeted therapies represent a promising therapeutic candidate for this devastating disease.

## 5 Materials and Methods

### 5.1 Study Participants and Cohort Design

All MMD patients were recruited from Beijing Tiantan Hospital, Capital Medical University. The diagnosis of MMD was based on the 2021 Japanese diagnostic criteria. According to the study design, four cohorts were established for different omics analyses: The mass cytometry (CyTOF) dataset was newly generated specifically for the present study and included 40 patients with MMD and 20 HCs. The scRNA‐seq and bulk‐seq cohort reused data generated by our group in a previous project [9]. The scRNA‐seq cohort comprised seven patients with MMD. For comparison, scRNA‐seq data from HCs were obtained from the publicly available Gene Expression Omnibus dataset GSE165080. The bulk‐seq cohort included 23 patients and six controls. An additional cohort of 50 patients with MMD and 25 HCs was included for untargeted serum metabolomic profiling. Their clinical characteristics are summarized in Tables S1–S4. Furthermore, to validate Treg characteristics in vitro, Tregs were sorted from seven MMD patients and seven healthy controls and subjected to flow cytometric analysis. Other detailed methods have been included in the Supporting Information Methods and Materials.

### 5.2 Statistical Analysis

Statistical analyses were performed using R (version 4.1.1) and Python (version 3.8). Depending on the data distribution and experimental design, appropriate statistical tests were applied, including Student's *t*‐test, Mann–Whitney *U* test, and one‐way ANOVA analysis. *p* value less than 0.05 was considered statistically significant. Detailed methods are described in the Supporting Information Materials.

## Author Contributions

Conceptualization, methodology, visualization, and Writing – original draft: Zhiyao Zheng and Qiheng He. Investigation: Zhiyao Zheng, Qiheng He, Xinyu Ma, Bojian Zhang, Junsheng Li, Chenglong Liu, Siqi Mou, Wei Sun, Xiangjun Shi, Wenjing Wang, Dong Zhang, and Peicong Ge. Experiments: Zhiyao Zheng and Xinyu Ma. Funding acquisition: Peicong Ge. Project administration: Wenjing Wang, Dong Zhang, Peicong Ge, and Jizong Zhao. Supervision: Dong Zhang, Peicong Ge, and Jizong Zhao. Writing, review, and editing: Zhiyao Zheng, Qiheng He, and Peicong Ge. All authors have read and approved the final manuscript.

## Funding

This study was supported by the National Natural Science Foundation of China (Grant No. 82301451), Capital Medical University for Excellent Young Scholars (B2421), “TianTan‐North Brain Young Talent” Program (BNJH‐13), and Health China BuChang ZhiYuan Public Welfare Projects for Heart and Brain Health (HIGHER2024011).

## Conflicts of Interest

The authors declare no conflicts of interest.

## Ethics Statement

Informed consent was obtained from all participants in the study, and the study protocol was approved by the Institutional Review Board of Beijing Tiantan Hospital (KY2022‐051‐02). The research adhered to the principles of the Declaration of Helsinki.

## Supporting information




**Supporting Material**: Multi‐omics insights into circulating T cell metabolic reprogramming and Treg dysfunction in moyamoya disease.

## Data Availability

The raw sequencing data reported in this paper have been deposited in the Genome Sequence Archive for Human (GSA‐Human) in the National Genomics Data Center, China National Center for Bioinformation/Beijing Institute of Genomics, Chinese Academy of Sciences (https://ngdc.cncb.ac.cn/gsa‐human). The single‐cell RNA‐sequencing data are available under accession number HRA005203, and the bulk RNA‐sequencing data are available under accession number HRA004479. The mass cytometry data have been deposited in the OMIX repository, China National Center for Bioinformation/Beijing Institute of Genomics, Chinese Academy of Sciences (https://ngdc.cncb.ac.cn/omix; accession number OMIX010775), and access may be requested through the repository's controlled‐access application procedure. The publicly available single‐cell RNA‐sequencing data from healthy controls and superficial temporal artery transcriptomic data used for external validation are available from the Gene Expression Omnibus under accession numbers GSE165080 and GSE249254, respectively. All other data supporting the findings of this study are available from the corresponding author upon reasonable request.
