# Supplementary material for: Multi‐Omics Insights Into Circulating T‐Cell Metabolic Reprogramming and Treg Dysfunction in Moyamoya Disease
Source: MedComm (2020). 2026 Oct 23;7(11):e71088. doi: 10.1002/mco2.71088 (PMC13693916; doi:10.1002/mco2.71088)
Supplement: Supplementary file 1 [file MCO2-7-e71088-s001.docx]

***Supplementary Material***

Multi-omics insights into circulating T cell metabolic reprogramming and Treg dysfunction in moyamoya disease

**Running title**: Multi-omics of Tcell and Treg Dysfunction in MMD.

Zhiyao Zheng^1,2,4,5†^, Qiheng He^1,2†^, Xinyu Ma^1^, Bojian Zhang^1^, Junsheng Li^1,2^, Chenglong Liu^1,2^, Siqi Mou^1,2^, Wei Sun^1,2^, Xiangjun Shi^6^, Wenjing Wang^3*^, Dong Zhang^7,8*^, Peicong Ge^1,2*^, Jizong Zhao^1*^

^1^Department of Neurosurgery, Beijing Tiantan Hospital, Capital Medical University; Beijing, China.

^2^Basic and Translational Medicine Center, National Clinical Research Center for Neurological Diseases, Beijing Tiantan Hospital, Capital Medical University; Beijing, China.

^3^Beijing Institute of Hepatology, Beijing Youan Hospital, Capital Medical University; Beijing, China

^4^Research Unit of Accurate Diagnosis, Treatment, and Translational Medicine of Brain Tumors, Chinese Academy of Medical Sciences and Peking Union Medical College; Beijing, China

^5^Department of Neurosurgery, Peking Union Medical College Hospital, Chinese Academy of Medical Sciences and Peking Union Medical College; Beijing, China

^6^Department of Rheumatology and Immunology, Beijing Tiantan Hospital, Capital Medical University, Beijing, China

^7^Department of Neurosurgery, Beijing Hospital, National Center of Gerontology, Beijing 100730, China

^8^Institute of Geriatric Medicine, Chinese Academy of Medical Sciences, Beijing 100730, China

* Correspondence: Wenjing Wang, No. 8 Xitoutiao, You'anmenwai, Fengtai District, Beijing, wangwenjing85321@ccmu.edu.cn;

Dong Zhang, No. 1, Dongdan Dahua Road, Dongcheng District, Beijing, zhangdong@bjtth.org;

Peicong Ge, No. 119, South Fourth Ring Road West, Fengtai District, Beijing, gepeicong@163.com.

Jizong Zhao, No. 119, South Fourth Ring Road West, Fengtai District, Beijing, zhaojizong@bjtth.org.

† These authors contributed equally to this work.

**The PDF file includes:**

Supplementary Methods and Materials

Supplementary References

Figure.S1 to S7

Table.S1 to S5

**Supplementary Materials and methods**

**Study participants and Cohort design**

All MMD patients were recruited from Beijing Tiantan Hospital, Capital Medical University. The diagnosis of MMD was based on the 2021 Japanese diagnostic criteria. According to the study design, four cohorts were established for different omics analyses: The mass cytometry (CyTOF) dataset was newly generated specifically for the present study and included 40 patients with MMD and 20 HCs; The scRNA-seq and bulk-seq cohort reused data generated by our group in a previous project^1^. The scRNA-seq cohort comprised seven patients with MMD. For comparison, scRNA-seq data from HCs were obtained from the publicly available Gene Expression Omnibus dataset GSE165080. The bulk-seq cohort included 23 patients and 6 controls. An additional cohort of 50 patients with MMD and 25 HCs was included for untargeted serum metabolomic profiling. Their clinical characteristics are summarized in Tables S1-S4. Furthermore, to validate Treg characteristics in vitro, Tregs were sorted from 7 MMD patients and 7 healthy controls and subjected to flow cytometric analysis.

**Sample processing and clinical data collection**

Peripheral blood samples were collected into EDTA tubes and kept on ice. All samples were processed within two hours to preserve cell viability. Peripheral blood mononuclear cells (PBMCs) were isolated by Ficoll-paque density gradient centrifugation and used for CyTOF, scRNA-seq, and bulk-seq. The serum was separated and stored for untargeted metabolomic analysis. Clinical data including demographics (age, sex), baseline data (heart rate, blood pressure, BMI), medical history (hypertension, diabetes, hyperlipidemia), and clinical presentation (hemorrhagic stroke, ischemic stroke, transient ischemic attack) were collected at admission. Two experienced neurosurgeons independently reviewed imaging to assess the Suzuki stage, posterior cerebral artery (PCA) involvement, and collateral grade.

**Single-cell immune and metabolic regulome profiling by mass cytometry**

CyTOF was employed to profile the immune and metabolic features of PBMCs at single-cell resolution. The antibody panel was designed based on previously published T-cell immune profiling and single-cell metabolic profiling (scMEP) protocols, as described in prior studies^2^. Cryopreserved PBMC samples were thawed and gently resuspended in a staining buffer. Cell viability was assessed using cisplatin staining. Subsequently, cells were stained with a panel of metal isotope-conjugated monoclonal antibodies targeting surface markers. Data acquisition was subsequently performed on a Helios mass cytometer (Fluidigm, USA).

**CyTOF data preprocessing and analysis**

Raw data preprocessing was performed using manual gating in Cytobank to exclude doublets, debris, and dead cells, and to isolate CD45^+^CD3^+^ T cells for downstream analyses. Based on previously reported workflows^3^, we conducted subsequent analyses using the CATALYST package in R, including normalization, clustering and visualization steps. CyTOF marker intensities were transformed to normalize signal distributions, which is critical for accurate and meaningful clustering. The resulting cell clusters were visualized using dimensionality reduction techniques, such as t-SNE by the TSNE package, and heatmaps generated using the ComplexHeatmap package. These visualizations were used to characterize annotated cell subsets and identify distinct biomarker expression patterns.

To further resolve T cell heterogeneity, CD4 and CD8A expression was used to distinguish CD4 T cells, CD8 T cells, double negative T cells (DNT), and double positive T cells (DPT). Within the CD4^+^ T cell population, unsupervised clustering identified six distinct subsets, including CD4^+^ naive (Tn, CD45RA^+^CCR7^+^CD45RO^-^), central memory (Tcm, CD45RO^+^CCR7^+^CD45RA^-^), effector memory (Tem, CD45RO^+^CCR7^-^CD45RA^-^), regulatory (Treg, CD25^hi^CD127^lo^CD152^+^), terminal effector memory (Temra, CD45RA^+^CCR7^-^CD45RO^-^), and exhausted (Tex, CD279^+^CD57^+^) T cells. CD8+ T cell population was divided in five subsets, including CD8+ Tn (CCR7^+^CD45RA^+^CD45RO^-^), Tem (CCR7^-^CD45RA^-^CD45RO^+^), Tactive (CD69^+^), Temra (CCR7^-^CD45RA^+^CD45RO^-^), and Tex (CD57^+^) cells.

**Single-cell library preparation and sequencing**

Single-cell RNA sequencing (scRNA-seq) of PBMCs was performed using the Chromium next GEM single cell 3’kit v3.1 (10x Genomics). Cells were encapsulated with barcoded gel beads in emulsion (GEMs), enabling mRNA capture and labeling with unique molecular identifiers (UMIs) and cell barcodes during reverse transcription. Then, libraries were constructed through second-strand cDNA synthesis, adaptor ligation, and amplification, enriching for 3′ transcript ends linked to barcodes and UMIs. All procedures followed the manufacturer’s protocol. Sequencing was performed on the Illumina NovaSeq platform.

**scRNA-seq data analysis**

Single-cell RNA-seq data from healthy donors were obtained from the Gene Expression Omnibus (GEO) under accession number GSE165080. Gene expression matrices were generated using Cell Ranger (v5.0.1) with the human reference genome GRCh38-1.2.0. Downstream analysis was performed using the Seurat R package^4^. During quality control, cells were filtered out if they met any of the following criteria: (1) UMI counts ≤ 500 or ≥ 15,000; (2) expression of ≥ 8,000 genes; (3) ≥ 10% mitochondrial UMI content; or (4) ≥ 1% hemoglobin UMI content. Potential doublets were identified and removed using DoubletFinder and manual curation. Data were normalized and scaled using the NormalizeData and ScaleData functions, and batch effects were corrected with Harmony integration^5^. Principal component analysis (PCA) was performed on the top 2,000 variable genes identified via the FindVariableFeatures function. Cells were clustered using the Louvain algorithm implemented through FindNeighbors and visualized using UMAP. For T cell subpopulation analysis, T cells were subset and reprocessed using the same workflow for normalization and clustering.

**Cell type annotation and T/NK subgroup identification**

Major cell identities were annotated following standard workflows. Briefly, various resolutions and numbers of principal components were evaluated during unsupervised clustering to optimize the number of cell clusters. The resulting UMAP plots, along with cluster-specific marker genes, were examined to determine optimal clustering. Major immune cell types were manually annotated based on marker genes identified using the FindAllMarkers function in Seurat, combined with well-established canonical markers. Specifically, gene signatures used for annotation included: CD14 and LYZ for myeloid cells; IRF7 and LILRA4 for plasmacytoid dendritic cells; FCGR3B, and G0S2 for neutrophils; GATA2 and CPA3 for mast cells; CD3D and NKG7 for T lymphocytes and natural killer cells; MS4A1, CD79A for B lymphocytes; and GNG11 and NRGN for platelets.

To further resolve T lymphocyte and natural killer cell heterogeneity, cells were categorized into four major populations based on canonical markers: CD4^+^ T (CD4 and CD40LG), CD8^+^ T (CD8A and CD8B), NK cells (FCGR3A and NCR1), and proliferating T cells (MKI67 and TOP2A). Subsequently, 13 distinct subclusters were identified, which were manually annotated using conventional lineage markers reported in the literature^6,7^.

**Differential expression and pathway enrichment analyses**

Differentially expressed genes (DEGs) between Tregs from MMD patients and healthy controls (HCs) were identified using the FindMarkers function in the Seurat package. Genes with a Benjamini–Hochberg-adjusted P value less than 0.05 and an absolute log2 fold change greater than 0.25 were considered significantly differentially expressed. Functional enrichment analysis was performed using the R package clusterProfiler to identify significantly enriched Kyoto Encyclopedia of Genes and Genome (KEGG) pathways^8^. Additionally, gene set enrichment analysis (GSEA) was performed to assess the relative enrichment of selected metabolic pathways, such as oxidative phosphorylation, glucose metabolism, small molecule metabolic pathways, and mitochondrial protein complexes.

**Trajectory analysis**

Trajectory analysis of CD4 T cell subgroups was reconstructed using the Monocle3 package^9^. Pseudotime analysis was performed to infer the temporal progression of CD4 T cell states alone the differentiation path. Besides, pseudotime values were extracted for each subgroup, and ridge plots were generated to visualize the distribution of subsets along the pseudotime axis, enabling the exploration of dynamic transitions and potential lineage relationships among CD4 T cell subgroups.

**Transcription factor regulon analysis**

To identify key transcription factors regulating distinct cell subgroups, we applied the pySCENIC algorithm^10^. For each regulon, activity scores were computed using AUCell, which estimates regulon enrichment at the single-cell level. To assess the cell type specificity of each regulon, we calculated regulon specificity scores (RSS) based on Jensen-Shannon divergence, quantifying the degree of cell type-specific regulatory activity across the identified CD4 T subpopulations.

**Cell-cell interaction identification**

Potential cell-cell communication networks were predicted using the CellChat R package^11^. We quantified ligand-receptor interactions between cell types to calculate interaction probabilities and assign signaling pathways. Only interactions with a P value less than 0.05 were considered statistically significant and included in downstream visualization and interpretation.

**Ligand activity and intracellular signaling inference**

Beyond predicting ligand-receptor interactions, NicheNet also integrates intracellular signaling pathways, enabling the inference of downstream pathway activation in receiver cells upon ligand stimulation^12^. To investigate regulatory signaling in Tregs, we defined MMD-related Treg genes as the set of genes upregulated in MMD compared to healthy controls. Based on this gene set, NicheNet was applied to identify the top-ranked ligands likely responsible for inducing the transcriptional changes. For each prioritized ligand, the top predicted target genes and the corresponding receptor molecules expressed in the Treg cell were inferred, providing a comprehensive view of potential ligand-receptor–target signaling axes.

**Single-cell metabolic flux analysis**

To investigate metabolic activity at single-cell resolution, we applied scFEA, a computational framework specifically designed to infer single-cell metabolic fluxes across a genome-scale metabolic network^13^. The "FLUX" scores predicted by scFEA reflect the relative activity of metabolic reactions, indicating how actively metabolites are converted within individual cells. Then, differential analysis was performed between disease and control groups to identify T cell-specific metabolic pathways. For each meta-reaction, we applied the Wilcoxon rank-sum test to compare flux scores, and calculated Cohen’s d to estimate the effect size of differences between groups^14^. Multiple testing correction was applied using the Benjamini–Hochberg method. Each reaction inherited the statistical results of its corresponding meta-reaction, and those with adjusted P values <0.05 were considered differentially active.

**Bulk RNA sequencing**

Total RNA was extracted from PBMC samples and subjected to library preparation and quality control following standard protocols. Sequencing was performed on the Illumina NovaSeq 6000 platform. Following quality control and alignment to the reference genome, gene expression quantification was performed using FeatureCounts. The resulting expression matrix was used for downstream analyses. Single-sample gene set enrichment analysis (ssGSEA) was calculated for comparison of MMD-related Treg score.

**Untargeted metabolomics analysis**

Serum samples were preprocessed and subjected to liquid chromatography–mass spectrometry (LC-MS) for metabolomic analysis. Chromatographic separation was performed using a HypersilGoldcolumn (C18), following a standardized protocol to ensure comprehensive metabolite identification and quantification. Metabolites were annotated using the KEGG, HMDB, and LIPID MAPS databases.

Data processing and normalization were performed using the metaX package^15^. Principal component analysis (PCA) and partial least squares discriminant analysis (PLS-DA) were conducted to explore sample distribution and identify key differentiating metabolites, with variable importance in projection (VIP) scores calculated for each feature. Statistical differences between groups were assessed using Student’s t-test. P values were adjusted for multiple comparisons using the Benjamini–Hochberg procedure. Metabolites were considered significantly different if they met the following criteria: VIP > 1, FDR < 0.05, and |log₂fold change| > 1.5.

**Tregs isolation**

Peripheral blood samples were collected from MMD patients and healthy controls. PBMCs were isolated from fresh venous blood by Ficoll-paque density gradient centrifugation. Isolation of Treg cells was performed using a human CD4^+^CD25^+^ T cell isolation kit (Miltenyi Biotec) with a MidiMACS separator unit, according to the manufacturer's instructions.

**Flow Cytometry**

Treg cells were stimulated with 3 µL of cell stimulation buffer in 1 mL of RPMI 1640 complete medium for 4 hours at 37°C in the dark. Cells were then stained with Zombie Aqua™ Fixable Viability dye (cat#423102, BioLegend) for 10 minutes at room temperature in the dark. Surface staining was performed using anti-human CD4 (cat#367-0047-42, Brilliant Ultra Violet737, eBioscience) and anti-PD-1 (cat#329920, Brilliant Violet 421, BioLegend). After fixation and permeabilization, intracellular staining was performed with anti-FOXP3 (cat#12-4776-42, PE, eBioscience) and anti-IL-17A (cat#512334, APC, BioLegend) for 60 minutes at room temperature in the dark. Isotype controls were used to define positive staining thresholds. Samples were acquired on a BD Fortessa X50 flow cytometer (BD Biosciences) and analyzed using FlowJo software (version 10.8).

**Statistical analysis**

Statistical analyses were performed using R (version 4.1.1) and Python (version 3.8). Depending on the data distribution and experimental design, appropriate statistical tests were applied, including Student’s t-test, Mann–Whitney U test, one-way ANOVA analysis. Univariate and multivariate binary logistic regression analyses were conducted to evaluate the relationship between Treg level and ischemic moyamoya disease. Variables with a *P* value < 0.05 in the univariate analysis were entered into the multivariate logistic regression model. *P*value less than 0.05 was considered statistically significant.

**Supplementary References**

1.	Ge P, Tao C, Wang W, et al. Circulating immune cell landscape and T-cell abnormalities in patients with moyamoya disease. *Clin Transl Med*. Apr 2024;14(4):e1647. doi:10.1002/ctm2.1647

2.	Hartmann FJ, Mrdjen D, McCaffrey E, et al. Single-cell metabolic profiling of human cytotoxic T cells. *Nat Biotechnol*. Feb 2021;39(2):186–197. doi:10.1038/s41587-020-0651-8

3.	Nowicka M, Krieg C, Crowell HL, et al. CyTOF workflow: differential discovery in high-throughput high-dimensional cytometry datasets. *F1000Res*. 2017;6:748. doi:10.12688/f1000research.11622.3

4.	Hao Y, Hao S, Andersen-Nissen E, et al. Integrated analysis of multimodal single-cell data. *Cell*. Jun 24 2021;184(13):3573–3587 e29. doi:10.1016/j.cell.2021.04.048

5.	Korsunsky I, Millard N, Fan J, et al. Fast, sensitive and accurate integration of single-cell data with Harmony. *Nat Methods*. Dec 2019;16(12):1289–1296. doi:10.1038/s41592-019-0619-0

6.	Chu Y, Dai E, Li Y, et al. Pan-cancer T cell atlas links a cellular stress response state to immunotherapy resistance. *Nat Med*. Jun 2023;29(6):1550–1562. doi:10.1038/s41591-023-02371-y

7.	Zheng L, Qin S, Si W, et al. Pan-cancer single-cell landscape of tumor-infiltrating T cells. *Science*. Dec 17 2021;374(6574):abe6474. doi:10.1126/science.abe6474

8.	Wu T, Hu E, Xu S, et al. clusterProfiler 4.0: A universal enrichment tool for interpreting omics data. *Innovation (Camb)*. Aug 28 2021;2(3):100141. doi:10.1016/j.xinn.2021.100141

9.	Cao J, Spielmann M, Qiu X, et al. The single-cell transcriptional landscape of mammalian organogenesis. *Nature*. Feb 2019;566(7745):496–502. doi:10.1038/s41586-019-0969-x

10.	Van de Sande B, Flerin C, Davie K, et al. A scalable SCENIC workflow for single-cell gene regulatory network analysis. *Nat Protoc*. Jul 2020;15(7):2247–2276. doi:10.1038/s41596-020-0336-2

11.	Jin S, Guerrero-Juarez CF, Zhang L, et al. Inference and analysis of cell-cell communication using CellChat. *Nat Commun*. Feb 17 2021;12(1):1088. doi:10.1038/s41467-021-21246-9

12.	Browaeys R, Saelens W, Saeys Y. NicheNet: modeling intercellular communication by linking ligands to target genes. *Nat Methods*. Feb 2020;17(2):159–162. doi:10.1038/s41592-019-0667-5

13.	Alghamdi N, Chang W, Dang P, et al. A graph neural network model to estimate cell-wise metabolic flux using single-cell RNA-seq data. *Genome Res*. Oct 2021;31(10):1867–1884. doi:10.1101/gr.271205.120

14.	Wagner A, Wang C, Fessler J, et al. Metabolic modeling of single Th17 cells reveals regulators of autoimmunity. *Cell*. Aug 5 2021;184(16):4168–4185 e21. doi:10.1016/j.cell.2021.05.045

15.	Wen B, Mei Z, Zeng C, Liu S. metaX: a flexible and comprehensive software for processing metabolomics data. *BMC Bioinformatics*. Mar 21 2017;18(1):183. doi:10.1186/s12859-017-1579-y

**Supplementary Figures**

**Figure S1**

**Figure S1. Gating strategy of T cells from mass cytometry (CyTOF).**

**Figure S2**

**Figure S2. Box plots showing the percentage of CD4 T and CD8 T cell subsets in moyamoya disease and healthy controls. (A).** subsets from CD4 T cells. **(B).** subsets from CD8 T cells. Mann-Whitney U test, p < 0.05, significantly different. Tn, naive T cell; Tcm, central memory T cell; Tem, effector memory T cell; Treg, regulatory T cell; Temra, terminal effector memory T cell; Tex, exhausted T cell; Tactive, active T cell.

**Figure S3**

**Figure S3.** Violin plots showing the log10-transformed expression levels of each metabolism marker in different CD8 T cell subsets in MMD patients.

**Figure S4**

**Figure S4**. **Violin plot showing the metabolic differences in CD4 T cell subsets.**

**(A)**．CD4^+^ naïve T cells; **(B)**. CD4^+^ central memory T cells; **(C)**. CD4^+^ terminal effector memory T cells; **(D)**. CD4^+^ exhausted T cells; Mann-Whitney U test, p < 0.05, significantly different.

**Figure S5**

**Figure S5.** **Violin plots showing the metabolic differences in CD8 T cell subsets.** **(A).** CD8^+^ effector memory T cells; **(B).** CD8^+^ exhausted T cells. Mann-Whitney U test, p < 0.05, significantly different.

**Figure S6**

**Figure S6.** **Landscape of scRNA-seq data in MMD patients and HCs. (A).** Uniform Manifold Approximation and Projection (UMAP) plot, color-coded by major cell types. **(B).** Dot plot showing the representative genes in each cell type. **(C).** UMAP plot showing the typical representative genes in each cell type, with expression levels distinguished by color. **(D).** Volcano plot showing the top 5 upregulated and downregulated genes in each cell type. **(E).** Bar plots showing the average cell type proportions in MMD patients and HCs. **(F).** Bubble plot illustrating the subclustering strategy for the T/NK cell population. **(G).** Bubble plot illustrating the subclustering strategy for the CD4+ T cell subset. pDC, plasmacytoid dendritic cells；NK, Natural Killer cell.

**Figure S7**

**Figure S7. Comparison of cellular communication between Treg cells and other cell types in MMD patients versus HCs, based on scRNA-seq CellChat analysis.**

**(A).** Communication weights between Treg cells and other cell populations in MMD patients compared to HCs. **(B).** Communication counts between Treg cells and other cell populations in MMD patients compared to HCs.

**Supplementary Tables**

**Supplementary Table1. Summary of clinical characteristics of participants in CyTOF cohort.**

MMD, Moyamoya disease; HC, Healthy control; TIA, Transient ischemic attack; PCA, Posterior cerebral artery;

| Characteristics | MMD patients  (N = 40) | HCs  (N = 20) |
| --- | --- | --- |
| **General Conditions** |  |  |
| Age (years; mean ± SD) | 38.5 ± 8.3 | 30.0 ± 5.3 |
| Female, n(%) | 20 (50.0) | 10 (50.0) |
| **Vascular risk factors, n (%)** |  |  |
| Smoking | 9 (22.5) | - |
| Drinking | 5 (12.5) | - |
| Hypertension | 12 (30.0) | - |
| Hyperlipidemia | 5 (12.5) | - |
| Diabetes mellitus | 1 (2.5) | - |
| **Clinical manifestations, n (%)** |  |  |
| Ischemic type | 20 (50.0) | - |
| Hemorrhagic type | 20 (50.0) | - |
| **Primary Symptom, n (%)** |  |  |
| Hemorrhage | 20 (50.0) | - |
| Infarction | 10 (25.0) | - |
| TIA | 10 (25.0) | - |
| **Suzuki stage, n (%)** |  |  |
| Stage 1-2 | 6 (15.0) | - |
| Stage 3-4 | 26 (65.0) | - |
| Stage 5-6 | 8 (20.0) | - |
| **PCA Involvement, n (%)** | 12 (30.0) | - |
| **Collateral Grade, n(%)** |  |  |
| 0-4 | 3 (7.5) | - |
| 5-8 | 28 (70.0) | - |
| 9-12 | 9 (22.5) | - |

**Supplementary Table2. Summary of clinical characteristics of participants in scRNA-seq cohort.**

| Characteristics | MMD patients  (N = 7) | HCs  (N = 7) |
| --- | --- | --- |
| **General Conditions** |  |  |
| Age (years; mean ± SD) | 41.9 ± 5.5 | 40.3 ± 5.8 |
| Female, n(%) | 3 (42.9) | 3 (42.9) |
| **Vascular risk factors, n (%)** |  |  |
| Smoking | 1 (14.3) | - |
| Drinking | 1 (14.3) | - |
| Hypertension | 2 (28.6) | - |
| Hyperlipidemia | 2 (28.6) | - |
| Diabetes mellitus | 0 (0.0) | - |
| **Clinical manifestations, n (%)** |  |  |
| Ischemic type | 4 (57.1) | - |
| Hemorrhagic type | 3 (42.9) | - |

**Supplementary Table3. Summary of clinical characteristics of participants in bulk RNA-seq cohort.**

| Characteristics | MMD patients  (N = 23) | HCs  (N = 6) |
| --- | --- | --- |
| **General Conditions** |  |  |
| Age (years; mean ± SD) | 31.1±8.5 | 30.0± 8.3 |
| Female, n(%) | 15 (65.2) | 4 (66.7) |
| **Vascular risk factors, n (%)** |  |  |
| Smoking | 1 (4.3) | - |
| Drinking | 3 (13.0) | - |
| Hypertension | 7 (30.4) | - |
| Hyperlipidemia | 3 (13.0) | - |
| Diabetes mellitus | 1 (4.3) | - |
| **Clinical manifestations, n (%)** |  |  |
| Ischemic type | 12 (52.2) | - |
| Hemorrhagic type | 11 (47.8) | - |
| **Primary Symptom, n (%)** |  |  |
| Hemorrhage | 11 (47.8) | - |
| Infarction | 12 (52.2) | - |
| TIA | 0 (0.0) | - |
| **Suzuki stage, n (%)** |  |  |
| Stage 1-2 | 8 (34.8) | - |
| Stage 3-4 | 11 (47.8) | - |
| Stage 5-6 | 4 (17.4) | - |
| **PCA Involvement, n (%)** | 2 (8.7) | - |

**Supplementary Table4. Summary of clinical characteristics of participants in untargeted metabolomic cohort.**

| Characteristics | MMD patients  (N = 50) | HCs  (N = 25) |
| --- | --- | --- |
| **General Conditions** |  |  |
| Age (years; mean ± SD) | 28.5 ± 8.0 | 39.0 ± 7.8 |
| Female, n(%) | 28 (56.0) | 14 (56.0) |
| **Vascular risk factors, n (%)** |  |  |
| Smoking | 11 (22.0) | - |
| Drinking | 7 (14.0) | - |
| Hypertension | 8 (16.0) | - |
| Hyperlipidemia | 2 (4.0) | - |
| Diabetes mellitus | 0 (0.0) | - |
| **Clinical manifestations, n (%)** |  |  |
| Ischemic type | 25 (50.0) | - |
| Hemorrhagic type | 25 (50.0) | - |
| **Primary Symptom, n (%)** |  |  |
| Hemorrhage | 25 (50.0) | - |
| Infarction | 12 (24.0) | - |
| TIA | 13 (26.0) | - |
| **Suzuki stage, n (%)** |  |  |
| Stage 1-2 | 11 (22.0) | - |
| Stage 3-4 | 32 (64.0) | - |
| Stage 5-6 | 7 (14.0) | - |
| **PCA Involvement, n (%)** | 13 (26.0) | - |

**Supplementary Table5. Univariate and multivariate logistic regression analysis of factors associated with ischemic onset in patients with moyamoya disease.**

| Characteristics | Univariate analysis | | | Multivariate analysis | | |
| --- | --- | --- | --- | --- | --- | --- |
|  | OR | 95%CI | P value | OR | 95%CI | P value |
| Age | 1.01 | 0.93 - 1.08 | 0.893 |  |  |  |
| Female | 2.25 | 0.63 - 7.97 | 0.209 |  |  |  |
| High Treg level | 4.33 | 1.15 - 16.32 | 0.030* | 5.95 | 1.31 - 27.12 | 0.021* |
| Smoking | 0.75 | 0.17 - 3.33 | 0.705 |  |  |  |
| Drinking | 4.75 | 0.48 - 46.91 | 0.182 |  |  |  |
| Hypertension | 4.64 | 1.02 - 21.00 | 0.047* | 6.65 | 1.19 - 37.07 | 0.031* |
| Hyperlipidemia | 4.75 | 0.48 - 46.91 | 0.182 |  |  |  |
| Diabetes mellitus | 0.00 | 0.00 - Inf | 0.994 |  |  |  |
